# Can echocardiographic particle image velocimetry correctly detect motion patterns as they occur in blood inside heart chambers? A validation study using moving phantoms

**DOI:** 10.1186/1476-7120-10-24

**Published:** 2012-06-06

**Authors:** Christian Prinz, Reka Faludi, Andrew Walker, Mihaela Amzulescu, Hang Gao, Tokuhisa Uejima, Alan G Fraser, Jens-Uwe Voigt

**Affiliations:** 1 Dept. of Cardiovascular Diseases, University Hospital Gasthuisberg, Catholic University Leuven, Herestraat 49, 3000, Leuven, Belgium; 2 Dept. of Biomedical Engeneering, Central Hospital, Västerås, Sweden; 3 Wales Heart Research Institute, School of Medicine, Cardiff University, Cardiff, United Kingdom; 4 Department of Cardiology, Heart- and Diabetes Center NRW, Ruhr-University, Bochum, Bad Oeynhausen, Germany; 5 The Heart Institute, Faculty of Medicine, University of Pécs, Pécs, Hungary

**Keywords:** PIV, Blood flow patterns, Echocardiography, Phantoms

## Abstract

**Aims:**

To validate Echo Particle Image Velocimetry (PIV)

**Methods:**

High fidelity string and rotating phantoms moving with different speed patterns were imaged with different high-end ultrasound systems at varying insonation angles and frame rates. Images were analyzed for velocity and direction and for complex motion patterns of blood flow with dedicated software. Post-processing was done with MATLAB-based tools (Dflow, JUV, University Leuven).

**Results:**

Velocity estimation was accurate up to a velocity of 42 cm/s (r = 0.99, p < 0.001, mean difference 0.4 ± 2 cm/s). Maximally detectable velocity, however, was strongly dependent on frame rate and insonation angle and reached 42 cm/s under optimal conditions. At higher velocities estimates became random. Direction estimates did depend less on velocity and were accurate in 80-90%. In-plane motion patterns were correctly identified with three ultrasound systems.

**Conclusion:**

Echo-PIV appears feasible. Velocity estimates are accurate, but the maximal detectable velocity depends strongly on acquisition parameters. Direction estimation works sufficiently, even at higher velocities. Echo-PIV appears to be a promising technical approach to investigate flow patterns by echocardiography.

## Introduction

Spectral- and color Doppler are powerful echocardiographic methods for imaging and quantifying blood flow velocities. However, they measure only the velocity component along the direction of the ultrasound beam and therefore cannot provide information on the direction or pattern of blood flow.

Recently introduced echocardiographic tracking algorithms for the assessment of myocardial motion stimulated the development of software which is capable of tracking image features in the blood pool in order to estimate blood motion in any direction within the image plane. This concept of Particle Image Velocimetry (PIV) [1], applied to contrast enhanced echocardiographic images [2,3], may allow not only to display and quantify blood flow velocity and direction, but may also provide new insights into typical cardiac flow patterns, such as vortices. The obtained information could then be used even to estimate energy dissipation in the flow field [4,5].

Previous studies have tested the software in comparison to other imaging techniques [6] or in virtual models [7] using flow patterns mimicking cardiac flow. So far, no detailed analysis of the influence of imaging geometry in combination with different acquisition settings has been performed.

In this study we used moving phantoms to investigate to what extent a newly developed echocardiographic PIV software (Omega Flow, Siemens, Mountain View, CA, USA*^a^) is able to correctly describe flow direction and velocity and to recognize flow patterns within the image.

## Methods

### Experiments

#### String Phantom

**Figure 1:**
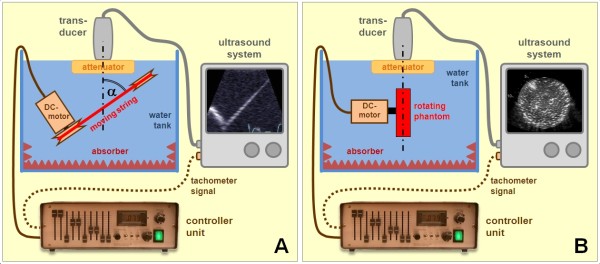
**Setup of the phantom experiments using string and rotating phantoms.** (**A**) String phantom to obtain a controlled linear motion pattern. Angle **α** indicates the deviation of the motion direction from the ultrasound beam direction. Note the controller unit with the connection to the ultrasound system for tachometer signals and the possibility for exact velocity measurements. (**B**) Rotating phantom to mimic a vortex-like structure with a comparable setup. See text for details.

In order to produce ultrasound images with a distinct speckle pattern with linear motion of known speed and direction, a commercially available high fidelity Doppler testing device (DP1, BBS Medical Electronic AB, Hägersten, Sweden) was used. It consisted of a loop of surgical silk tautened between two pulleys. The position of the pulleys could be adjusted in a way that the insonation angle at which the string was imaged could be changed between 0 and 90 degrees (Figure 1A) [8,9]. A watertight DC-motor, controlled by an external control unit, drove one of the pulleys. The control unit allowed to set the rotational speed of the motor to a constant value or to program repetitive sequences of velocity profiles [8,9]. A tachometer was directly attached to the motor shaft and allowed to measure the actual rotational speed with high fidelity. The tachometer signal was also available as a proportional analogue output which was fed into a physio-channel of the ultrasound system and digitally stored together with the image data. The speed of the string was calculated by multiplying the known circumference of the pulley with the rotational speed of the motor.

#### Rotating phantom

The rotating phantom was used to test both, the flow recognition at different angles as well as the vortex recognition algorithm. A flat cylinder of agar of 3 cm thickness and 10 cm in diameter was cast on a disc of acrylic glass. Holes were punched in the agar with a fine needle in a way that an evenly distributed pattern of distinct speckles was obtained when the phantom was imaged. The disc with the agar phantom was directly fitted to the shaft of the DC-motor of the same testing device [10] (Figure 1B). The tangential velocity of a certain point in the agar phantom could be calculated by multiplying its distance from the motor axis with 2π and the rotational speed of the motor.

#### Setup and Imaging Protocol

Phantoms were submerged in a rubber damped tank which was filled with degassed tap water. The ultrasound transducers were placed in a tripod and positioned just below the water surface. A regular attenuator, as used to avoid near field artefact in soft tissue imaging, was placed under the probes (Figure 1A and 1B).

The string phantom could be turned to obtain different insonation angles. Care was taken to center the ultrasound transducer over the tilt axis of the string phantom which was then imaged at a depth of approximately 7.5 cm. Similarly, the rotating phantom was positioned in the center of the image with its rotational axis at a depth of approximately 9.5 cm.

Images were collected with high-end ultrasound systems (Siemens Acuson SC 2000, Siemens Medical Solutions, Mountain View, USA, probe: 4V1c-S, 1.0-4.0 MHz; GE Vivid E9, Vingmed Ultrasound, Horten Norway, probe: M3S, 1.5-4.0 MHz; Philips iE33, Philips Ultrasound, Bothell, WA, USA, probe: S5-1, 1.0-5.0 MHz). Image loops of at least three seconds were digitally stored at three different gain settings (−10, 0, +10 dB) to allow the selection of the optimal image for subsequent off-line analysis. The optimal image was selected, if already in part a pure visual tracking of specific speckle-patterns was possible.

In order to investigate the influence of frame rate, three different acquisition settings resulting in comparable frame rate ranges (38–44 fps, 56–62 fps and 74–81 fps) were used in all ultrasound machines.

**Figure 2:**
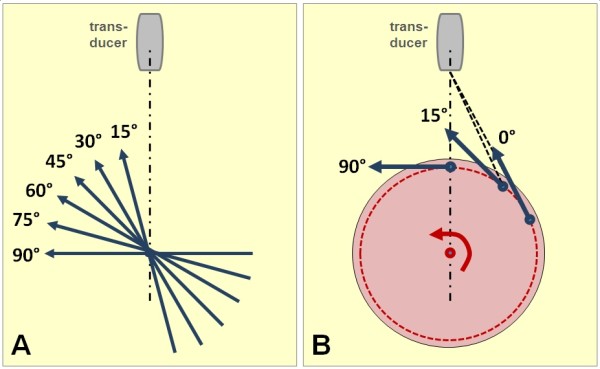
**Acquisition and analysis of phantom data.** (**A**) In the string phantom, the direction of the string motion was set during acquisition in steps of 15° relative to the ultrasound beam. (**B**) In the rotating phantom, different insonation angles could be obtained during post-processing by placing the sample volume of the post-processing program at different pre-defined points around the circumference of the phantom. See text for further details.

To investigate the influence of axial vs. lateral resolution, the insonation angle of the string was changed between 15 and 90 degrees in steps of 15 degrees (Figure 2A). Similarly, measurements at different points of the rotating phantom also allowed to investigate changing insonation angles (Figure 2B).

For determining the accuracy of velocity and angle estimates, the rotating phantom was driven at different continuous speeds resulting in circumferential velocities between 5 and 400 cm/s.

In order to investigate the limits of tracking, both the string and the rotating phantom were driven using a ramp pattern with increasing string speed up to a varying plateau between 10 and 100 cm/s. The ramp was repeated 60 times per minute.

### Postprocessing

#### Particle Image Velocimetry (PIV)

**Figure 3:**
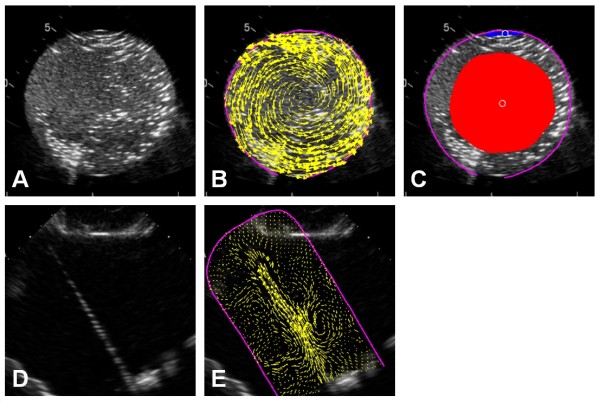
**Data processing in the flow tracking software.(A)** Echo image of the rotating phantom. (**B**) After tracing the circumference of the phantom (purple line), the software tracks speckles inside this area and displays the estimated flow by means of arrows (yellow) (in this specific figure a velocity of 20 cm/s was used). (**C**) In a further step, the software defines the biggest continuous area of vortical flow as “vortex” which is marked with a red or blue patch depending on the rotation direction. The white circle indicates the detected vortex center. Note that the patch is smaller than the phantom – see text for details. (**D**) Echo image of the string phantom (**E**) After tracing the circumference of the phantom (purple line), the software tracks speckles inside this area and displays the estimated flow by means of arrows (yellow) (in this specific figure also a velocity of 20 cm/s was used).

Echocardiographic image loops were processed offline using a dedicated prototype software (Omega Flow Version 2.3.1.). Since the software is designed for ventricular flow analysis, the first analysis step is to trace and track the endocardial border of the cavity of interest. For the string phantom image loops, we drew a virtual endocardial contour in ca. 2 cm distance to the string in the center of the image. In the rotating phantom image loops, the contour was just enveloping the phantom. In both, the endocardial tracking function of the program was switched off in order to keep the contour stable (Figure 3 A and D).

In a second step, the circumscribed area of interest is analyzed by the feature tracking algorithm of the program which provides a matrix of instantaneous local flow velocity vector estimates based on the tracking of the moving scatterers (Figure 3B and E). Generally, the highest possible matrix density setting was used (approx. one vector estimate per 8*8 image pixels). For evaluating the vortex recognition in images from different machines, medium and low matrix density settings were tested as well.

The velocity matrix is then used to calculate the local in-plane vorticity. The software provides an estimate of the position and size of the dominant clockwise and counterclockwise vortices within the circumscribed region. For the latter, the threshold for including detected vortical flow in the main vortex area could be set as 25%, 50%, 75% or 100% of the maximum heartbeat-averaged steady-streaming field (Figure 3C). We defined the biggest continuous area of vortical flow as steady-streaming field.

Both the velocity matrix as well as the vortex characteristics were exported for further analysis [4,5].

#### Data analysis

**Figure 4:**
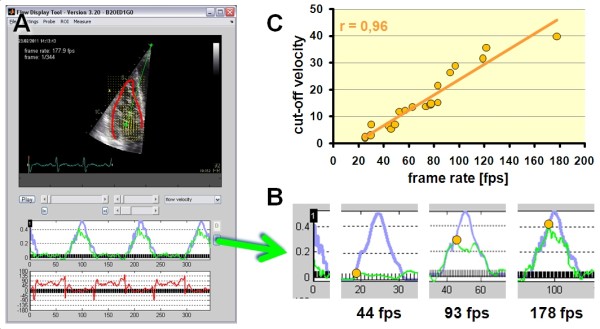
**Analysis of flow estimates.** (**A**) With a dedicated software (dFlow, JUV, Catholic University Leuven), single cells of the vector matrix were selected. The example shows a human right ventricle with the sample volume at the tips of the tricuspid valve. The blue curve in the middle panel shows the true inflow velocity profile as it was imported from a PW-Doppler trace from exactly the same position. The green curve shows the tracking derived velocity estimate in the selected matrix cell of the flow vector field. The red curve in the lower panel shows the tracking derived direction estimate (in this case, zero is defined as direction towards the transducer). (**B**) Examples of tracking results of the same tricuspid inflow profile, acquired at frame rates of 44, 93 and 178 frames per second. The yellow dots indicate the highest correct velocity estimate (cut-off velocity). (**C**) Relation of measured cut-off velocities vs. acquisition frame rates in tricuspid and mitral valves. Note the high frame-rate dependence of tracking results.

For evaluation of the accuracy of the estimates of flow direction and velocity as well as for determining the maximally measurable flow velocity (cut-off velocity) we used a dedicated, custom made, MATLAB based software tool (dFlow, JUV, Catholic University Leuven) which allowed to extract curves of regional velocity and motion direction from each point of the velocity matrix and to compare those to the true velocity profiles which had been stored as synchronous physio-trace with the image data (see example in Figure 4A).

For string phantom data, the sample volume of the dFlow program was placed in the midline of the image sector directly on the string. In the rotating phantom clips, the sample volume was positioned in a way that different insonation angles could be investigated. The distance of the sample volume to the phantom center of rotation was measured in the image in order to scale the known velocity profile of the phantom to the true velocity in sample position (Figure 2 A and B).

### Proof of principle in human hearts

For a proof of principle, we collected contrast enhanced echo data from routine patients of the clinical echo lab in Leuven who had good apical echo quality and normal cardiac structure and function on the routine echocardiogram. The study had been approved by the ethical committee of our institution and all patients had given their informed consent prior to the examination. 20 echocardiographic image loops of good quality but varying frame rate (25 – 179 fps) showing the three- or four chamber view of human hearts after a bolus injection of 0.2 ml SonoVue were analyzed with the PIV software as described above. Flow structures were displayed and cut-off velocities were determined by comparing the tracking derived flow velocity component towards the probe at the tip of the mitral or tricuspid valve leaflets to pulsed wave Doppler traces from the same position during ventricular filling (Figure 4).

### Statistical analysis

#### Determination of the cut-off velocity

**Figure 5:**
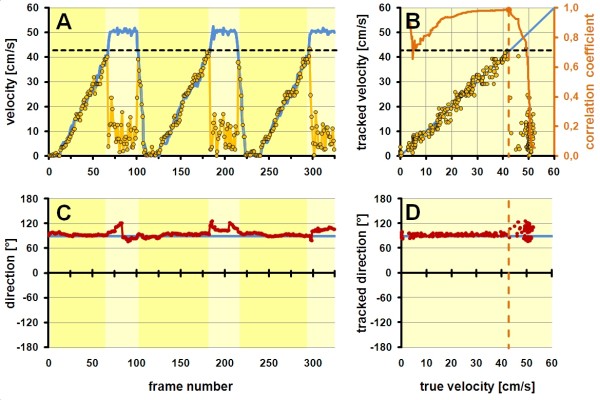
**Example of the determination of the cut-off velocity and direction estimation error in a typical acquisition from the phantom.** (**A**) The blue curve indicates the true phantom velocity at the sample position as determined from the tachometer signal of the phantom (please see Figure 1). The yellow dots indicate velocity estimates from the tracking software. Note that the almost ideal tracking up to a certain velocity (yellow shaded areas) and the complete failure of tracking above this velocity (“cut-off velocity”). (**B**) Statistical approach for the objective determination of the cut-off velocity. Same tracking data as in (**A**) are now displayed vs. the true velocity. The failure of tracking is clearly visible as marked deviation from the line of agreement. Data pairs were included stepwise with increasing true velocities and the correlation coefficient was calculated (orange line). This coefficient reaches its maximum at the cut-off velocity and drops dramatically afterwards. This maximum was automatically detected and used as definition of the cut-off velocity (orange dashed line). (**C**) The red line represents the flow direction estimate of the tracking software from the same sample position as in (**A**). The true motion direction is indicated by the blue line. Note the relatively stable direction estimates even when velocity estimation is failing due to too high velocities. (**D**) Same data as in (**C**) shown in relation to the true velocity of the phantom. Note the clear, but mild deterioration of tracking above the cut-off velocity (orange dashed line).

When using a ramp profile exceeding the highest trackable velocity, velocity estimates of the PIV software followed the true velocity profile up to a certain point and showed then a random pattern (see example in Figure 5A). To determine this cut-off velocity in an objective way, a statistical approach was used: In a linear regression model with the true velocities on the x axis and the estimated velocities on the y axis, the fit of a linear regression line crossing the coordinate origin was calculated for a stepwise inclusion of data pairs of increasing true velocities. The true velocity at the step with the maximum fit served as cut-off velocity (see example in Figure 5B).

#### Determination of the accuracy of the velocity estimate

In the string phantom, velocity estimates from the center of the image were compared to the known velocity using the method of Bland and Altman [11]. In the rotating phantom, error histograms of the entire flow field were calculated. In ramp profiles, data below and above the cut-off velocity were considered separately.

#### Determination of the accuracy of the direction estimate

In the string phantom, a direction estimate from the center of the image was analyzed. A deviation from the true angle of less than ± 10° was arbitrarily accepted as accurate and the relative frequency of accurate measurements was determined in relation to the true velocity of the phantom. In the rotating phantom, the deviation of the angle estimate from the truth was analyzed using error histograms for the entire flow field. In ramp profiles, data below and above the cut-off velocity were considered separately (see example in Figure 5 C and D).

In general, continuous variables are presented in the following as mean ± standard deviation (SD). Linear regression was used to investigate the relation between two parametric variables. Continuous measurements were compared by Spearman correlation and a Bland-Altman-Analysis. A two-tailed p value of < 0.05 was considered significant. Statistical analyses were performed with MATLAB (dito) and the PASW™ software (SPSS Inc., Chicago, Illinois).

## Results

The echocardiographic PIV software was able to follow the moving speckle pattern produced by the phantoms. Only acquisitions from the string phantom at 90 degree insonation angle were not analyzable for technical reasons, since the speckles produced by the surgical silk merged to a continuous white line. Estimates of motion velocity and direction were related to the true motion of the phantoms as reported below. The rotating phantom was properly recognized as a vortical structure.

### Velocity estimates

Accuracy of PIV velocity estimates was found to depend strongly on acquisition settings.

**Figure 6:**
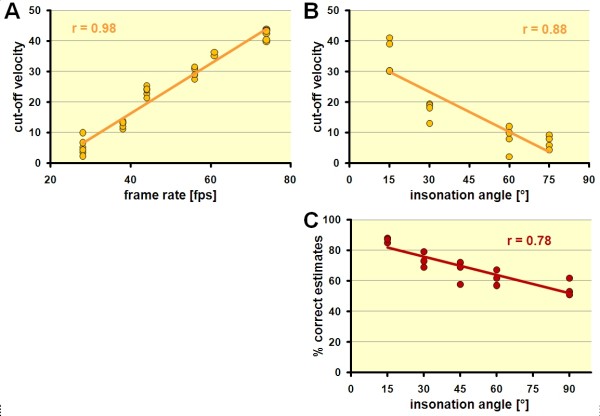
**Influence of imaging parameters on flow tracking.** (**A**) The cut-off velocity shows a clear frame rate dependence (see text for details). (**B**) The cut-off velocity is also dependent on the insonation angle. Best results are achieved when motion is close to the ultrasound beam direction (please see Figure 2). (**C**) Likewise, the accuracy of the flow direction estimate is insonation angle dependent. Best results are achieved when motion is close to the ultrasound beam direction (please see Figure 2).

In both string and rotating phantom, we found a significant correlation between cut-off-velocity and frame rate (string phantom: r = 0.997, p < 0.01 and rotating phantom: r = 0.5, p < 0.001, resp.) (Figure 6A).

Using the string phantom, we could further show a significant correlation between cut-off-velocity and insonation angle with (r = −0.88, p < 0.001) (Figure 6B). The highest cut-off velocity was reached when the motion direction was close to the beam direction and lowest, when close to perpendicular to it.

**Figure 7:**
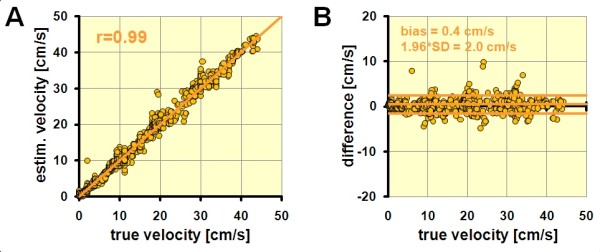
**Accuracy of velocity estimates below the cut-off velocity.** (**A**) The correlation between tracking derived velocity estimates and true velocity is excellent (see text for details). (**B**) Bland-Altman-plot of the same data. Note the low bias and variation.

Comparing all settings, the highest velocity which could be tracked (cut-off velocity) was 42.8 cm/s in our study (see example in Figure 5). PIV was always able to estimate velocities below the cut-off velocity with good accuracy. The correlation between measured and true velocity was on average r = 0.996 (p < 0.001) (Figure 7A). Bland-Altman analysis revealed only a minor bias of 0.4 ± 2 cm/s without systematic error (Figure 7B). Above the cut-off-velocity, velocity estimates became unreliable and underestimated the true velocity with high variability.

#### Direction estimates

Direction estimates proved to be less sensitive to changing acquisition settings. We found no significant relation between the frequency of accurate direction estimates and frame rate. However, we noticed a relation to the insonation angle. The error of the direction estimate was lowest when the motion was close to the beam direction (Figure 6C).

Estimates became already accurate at very low velocities (above 4–8 cm/s) and remained stable up to the cut-off-velocity. Above the cut-off-velocity, estimates were less, but still sufficiently accurate (see example in Figure 5C and 5D). Under optimal conditions (low insonation angle in string phantoms), direction estimates deviated less than ±10° from the true direction in 86.7 ± 1.5% of all frames.

#### Pattern recognition

The PIV software was able to identify the rotating agar phantom as vortex (see example in Figure 3C). The vortex center was accurately identified with a minor offset of < 5.5 mm. Interestingly, the size of the indicated vortex region varied with the rotational speed of the phantom. The identified vortex had at maximum an approximate diameter of 2/3 of the region of the phantom in which the tangential velocities were still below the cut-off velocity for tracking. In general, the accuracy of the detection of the vortex center (p = 0.5) and the estimation of vortex size (p = 0.4) did not depend on the density settings of the flow vector field.

#### Intermachine comparison

The maximally detectable velocity (cut-off velocity) in the different high-end ultrasound machines was comparable (44 cm/s, 42 cm/s, and 42 cm/s, n.s.). We found further no significant differences for direction estimation in the different machines (80–90% of all frames within the ±10° range of the true direction for all machines).

The error for the vortex center detection was comparable (4.7 ± 3.5 mm, 5.5 ± 3 mm and 4.0 ± 3.1 mm). Acquisition and post-processing settings had no relevant impact on the accuracy of the vortex pattern recognition in image loops from the different machines.

#### Human data

**Figure 8:**
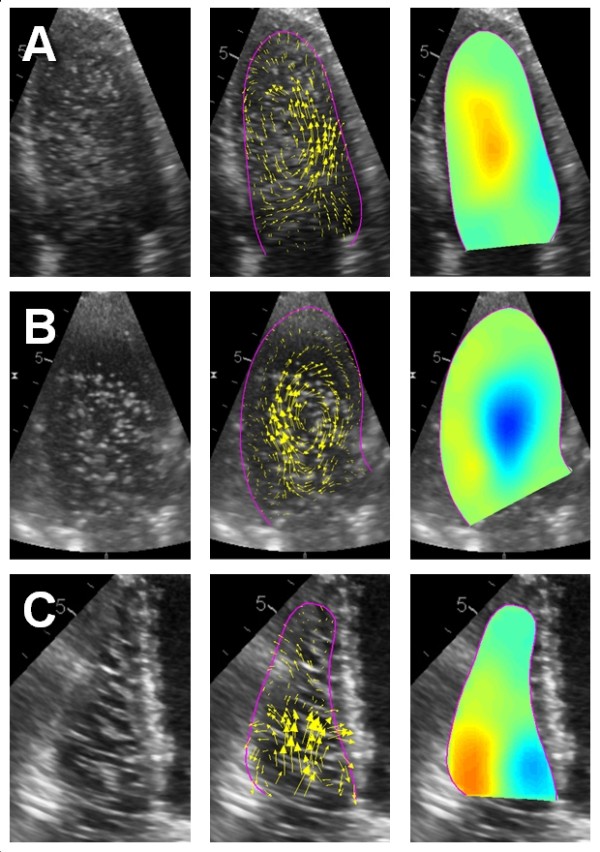
**Examples from in vivo imaging.** The panels on the left show the contrast-enhanced echo image. The middle panels show the traced cavity border (purple line) and the tracking derived flow estimates (yellow arrows). The right panels show the colour-coded instantaneous normalized vorticity. All images are taken at the end of rapid filling. (**A**) Four-chamber view. Note the big counter-clockwise vortex formation in the center of the ventricle (orange colour). (**B**) Three-chamber view. In this view, the same vortex structure is shown as a clockwise rotation direction (blue colour). (**C**) Right ventricle. In a normal RV, only minor vortex formation around the tips of the tricuspid valve is found (orange and blue area at the RV base).

We noted a counterclockwise vortex in the four-chamber-view of the LV during diastole in all patients (Figure 8A). In the LV three-chamber-view, a clockwise rotation of the blood was seen (Figure 8B). In the RV, minor diastolic vortices were noted just above the tricuspid valve (Figure 8C).

In concordance with the phantom experiments, the cut-off velocity proved to be linearly dependent on frame rate (r = 0.94, p < 0.01) (Figure 4C).

## Discussion

Flow can be described as laminar, vortical or turbulent. Typically, laminar flow is observed in straight blood vessels, vortex formation occurs in heart chambers, and turbulence may arise from stenotic valves. Vortex and recirculation may also occur in vessel bifurcations and turbulences in vessel stenoses [4,12-17]. A vortex is a structure able to store kinetic energy while turning. Turbulent flow, characterized by chaotic appearance of vortical structures on different scales, however, leads to rapid dissipation of kinetic energy. Flow patterns may therefore be relevant for an energy-efficient cardiac function and their non-invasive detection by echocardiography may be of clinical interest.

In this study, we used an in-vitro model of string and rotational phantoms to mimic laminar and vortical flow patterns. Our results indicate that PIV-echocardiography is able to estimate flow velocities and direction of moving speckle patterns in these in-vitro models. Accuracy of velocity estimation was strongly dependent on acquisition settings, but turned out to be sufficiently accurate for lower velocities below a certain cut-off velocity. The estimation of flow direction was less sensitive to changing acquisition settings and sufficiently accurate. The mimicked vortical flow pattern was recognized, even in varying settings with different ultrasound machines.

### Velocity estimates

Both the existence of a cut-off velocity and its frame rate dependence, can be explained with the underlying tracking algorithm: a speckle pattern can only be recognised and found back again in the following frame if its displacement is less than the maximum search distance of the algorithm. If the flow velocity is too high or the frame rate too low, speckle patterns move further than this and the tracking fails. In general terms, the cutoff velocity is about the length of the search window (in pixels) * pixelsize * framerate. In a normal echocardiographic study of a heart chamber (pixelsize = 0.25 mm and frame rate 50frames/sec) using a 32x32 window, this means a cutoff velocity of 0.4 m/s. This theoretical assumption is in good agreement with our measurements with a maximal measured cut-off velocity of 0.44 m/s.

This low cut-off velocity is a current limitation of the technique. It will be regularly exceeded already under physiologic conditions in the inflow region of the ventricle. For the remaining areas of the ventricle or the atria [18], however, it will be fully sufficient. Besides that, pattern recognition will be less affected, since our data demonstrate that direction estimates are still reasonable above the cut-off velocity.

The angle dependency of the cut-off velocities as seen in the practically one-dimensional string phantom can be best explained by the changing image resolution in motion direction when the phantom is tilted: a good resolution in-line with the beam results in higher cut-off velocities while the lower image resolution across beams delivers worse results.

#### Direction estimates

Surprisingly, flow direction estimates remained reasonably accurate even above the cut-off velocity (Figure 5C and 5D). We hypothesize that this phenomenon could be explained if the correlation of speckle patterns between frames is higher in the direction of flow than it is perpendicular to flow.

#### Pattern recognition

The displayed vortex size was dependent on the rotational speed of the phantom, but always smaller than the phantom size. While the former is explained by the loss of tracking in the outer regions of the phantom which exceed the cut-off velocity, the latter effect is due to the particularities of the rigid phantom. In contrast to a fluid vortex in the heart which has centrifugally decreasing angular velocities and therefore its highest vorticity in the center, the rigid phantom shows constant angular velocities in all parts which results in highest vorticity values at the outer edge. Under such circumstances, the implemented algorithm - which looks for a compact vorticity region above a certain threshold percentage of the heart beat averaged maximum vorticity – results in a radius of the displayed vortex (r_v_) proportional to the phantom radius (R): $r_{v}\approx \frac{R}{\sqrt{2}*threshold}$ (personal communication with G. Pedrizzetti, University of Trieste, Italy).

#### Intermachine Comparison

We observed no relevant difference between the tracking results from image loops acquired with the different high-end ultrasound machines. This may not fully reflect clinical reality since our phantoms, providing close-to-ideal imaging conditions, did not challenge the imaging capabilities of the different systems. Our results show clearly, however, that care must be taken to preserve original image resolution and frame rate during DICOM-conversion.

#### Human Data

Our findings indicate that requirements for image acquisition as revealed by our phantom experiments, apply fully to the clinical settings. Therefore, care must be taken to have ideal imaging conditions, in particular, good spatial resolution and frame rates as high as possible. Since such conditions can not always be found in clinical patients, a limited feasibility of the approach in a routine setting must be expected.

#### Limitations

In this study we used phantoms for the validation of PIV echocardiography which provided a defined two-dimensional pattern of speckle motion. In clinical practice, out-of plane motion of contrast speckles may lead to inaccuracies in the assessment of complex vortex structures in human heart chambers [19,20]. Interpretation of 2D tracking results must always consider, that at best the in-plane component of a 3D flow structure can be assessed. Three-dimensional echocardiography may help to overcome this limitation, but volume rates and spatial resolution of current machines are not sufficient for flow tracking in humans.

Our phantoms provided close-to ideal image quality without artefacts and homogeneous distribution of speckles. Less favorable scanning conditions in cardiac patients and inhomogeneities in contrast distribution may lead to tracking problems and subsequent misinterpretations of the flow field.

In our study, speckle patterns were generated by solid bodies, while in the clinical application they are generated by a non-Newtonian fluid. Together with the spatial character of fluid vortices, this may limit applicability of the model to the in vivo situation.

Given the underlying methodology, it must be assumed that tracking results do also depend on other imaging parameters, such as speckle distribution and density [20]. Therefore the sensitivity of the algorithm to the number of speckles per unit area was not evaluated. The current setup was not suited to investigate this relation. Furthermore, it is unclear how the software determines the periphery of vortex structures, since velocities are gradually declining when increasing the distance from the center. Further studies are needed to address those questions.

#### Clinical perspective

Particularly in diastole, kinetic energy of the blood entering the ventricle should be stored and the blood flow should be re-directed for ejection with a minimum of energy loss. A vortex structure appears ideally suited for this and may help to maximize ventricular efficiency. Therefore, echocardiographic PIV can provide new insights into diastolic function and dysfunction of the heart chambers. Further, the method might help to better understand the effect of regional myocardial dysfunction, e.g. after myocardial infarction, or help to optimize surgical interventions, such as valve replacements.

## Conclusion

The new method of echocardiographic particle image velocimetry (PIV) appears feasible on high quality, high frame rate DICOM images from different ultrasound machines. Accuracy of tracking results depends strongly on acquisition settings, image quality and absolute flow velocities. While velocity estimates are only accurate below a certain cut-off velocity, motion direction estimates appear more robust, even at higher velocities. This may allow a correct interpretation of flow patterns (relative vorticity) even if absolute flow velocity estimates become inaccurate. The demand on high quality, high frame rate image data appears as a current limitation to the use of the method in the clinical setting.

## Endnote

^a^At the time point of study. Currently, the software is marketed under the name “HyperFlow”, AMID, Trieste, Italy.

## Competing interests

The author(s) declare that they have no competing interests.

## Authors’ contributions

CP and RF collected and interpreted the data, carried out the statistical analysis and wrote the manuscript. AW collected and interpreted the data. MA, HG and TU collected and interpreted the data and contributed to the manuscript. AGF collected data, was involved in designing of the study, the drafting of the manuscript and revising it critically for important intellectual content. JUV was involved in the design of the study, interpretation and collection of the data and writing of the manuscript. All authors read and approved the final manuscript.
